# Notes and News

**Published:** 1892-10-22

**Authors:** 


### Notes and News.

Collected and Communicated.

A meeting of that association which calls forth so much sympathy, the Royal National Lifeboat Institution, was held last Saturday, when Mr. Macara, the most zealous and active Chairman of the Manchester branch, had to report much good work effected during the past year. The "Lifeboat" Saturday is in a fair way of being widely established. Many new lifeboats have been added to the fleet, and the Committee have had the pleasure of making many awards for gallant conduct on the part of the crews. The Hearts of Oak Benefit Society have presented the institute with £700 for the purpose of equipping a boat especially to be named the "Heart of Oak." The name is a happy one, and those who participate in the generous gift will watch the noble career of their boat with interest.

Droitwich adds a new feature to the list of medical charities in the hospital, or home, we would prefer to call it, just opened through the generosity of Mrs. Dyson Perrins, of Malvern. This new institution replaces the two cottages that up to now have served poor patients as a home when seeking relief from rheumatic ailments by the use of the Droitwich brine baths. These cottages were quite inadequate for the purpose. The new home is of brick, faced with terra-cotta, and roofed with slates. The ground floor is occupied by two women's sleeping apartments, containing five and three beds respectively, and day rooms for men and for women divided by a moveable partition. The kitchen joins the day rooms, and on this floor the matron's room is situated. The men's bed-rooms are at the other end of the building; there are separate entrances and staircases. On the upper floor are more patients' bedrooms, and also rooms for the staff. Both floors are provided with bath-rooms and other offices. Thirty-two patients can be accommodated, and admission is by a nomination through a subscriber whose subscription of one guinea covers a fortnight's residence, the patient paying five shillings a week beyond this. It is found that proper accommodation and diet cannot be provided for a less sum.

A professor in Vienna, who has recently died, interrogated a medical student in an examination, "What are the functions of the spleen?" After some reflection the student replied that he could not recollect. "What! miserable man, you who alone knew have forgotten," cried the Professor.

The decision to close the Addenbrooke's Hospital entirely for the reconstruction of drains, reserving only a temporary ward in the Anatomical Museum for accident cases, is causing some consternation now that the time approaches to carry out the works. Eight weeks without a hospital is a considerable period at this time of the year, and no doubt this point will be reconsidered before it is too late. At the quarterly Court held last week, however, nothing respecting the part or entire closing of the institution was discussed. Mr. Eaden Lilley has placed an excellent house, rent free, at the disposal of the authorities as a residence for nurses and domestic staff during the alterations.

It is bad news that scarlet fever still shows an increase in the metropolis. It is hoped that during the present week all the beds at Tottenham, beyond the 180 at present occupied, will be ready for use. There can be no doubt that once safely housed from a possibility of spreading infection in crowded homes, the number of cases would rapidly diminish; but matters having got so far beyond control, it will be some time before the down-hill course will end. These are bad times for the Asylums Board. Whilst in the midst of the difficulty of fever cases, they are brought to task for not possessing small-pox wards with ventilation of a special kind. Now the question of compensation has arisen again, and householders in the vicinity of their hospitals are crying out at the diminished value of their property. Alas! in all these cases the Board find they can do nothing, but doing nothing is not being made easy for them.

In referring to a short paragraph respecting the Salisbury Infirmary, which appeared recently in our pages, a Governor takes exception to our remarks respecting the present Matron, inasmuch as in praising her he considers we do the late Matron, Miss Morris, an injustice. He says, to her able management the diminution in household expenditure, which we spoke of, was due, and not to Miss Carvosso. If the gentleman in question will refer to the Chairman's speech at the recent annual meeting of the infirmary, he will find our remarks justified, and will see that the Chairman pointed out that "the Matron had, since her tenure of office, reduced the expenses very considerably in the departments over which she had control." This is an official statement, and we imagine that the Governor who writes cannot question its accuracy. Besides, the figures quoted by a Governor include items over which the Matron has no control. Quite recently we visited the infirmary personally, and found it, owing to Miss Carvosso's capable management, about the best administered county infirmary that we are acquainted with. Knowing this, and that Miss Carvosso combines in her person the duties of matron and housekeeper also, we still consider that those Governors who opposed a rise in salary to so excellent an officer made a very great mistake, which we hope will be promptly remedied.

